# Bromide of Potassium

**Published:** 1869-08-15

**Authors:** 


***Bromide of Potassium.***

Doctor August Maréchal, in summing up the new facts lately elicited, favorable to the treatment of epilepsy with this remedy, commends most highly the syrup of bromide of potassium, exempt from iodine, prepared by Mr. Henry Mure (Pont St. Esprit, Gard), and assigns the presence of iodine, or chlorine in the salt, as the cause of the gastric irritation, diarrhœa, loss of appetite, and emaciation sometimes consecutive upon the use of the bromide. In the same connection he quotes M. Prof. Sée (of la Charité), who asserts that "a new era has dawned for epilepsy; certain cases may be modified, others may be completely cured."

M. Becoulet, adjunct physician for the Hospital for the Insane, at Auxerre, says: "Bromide of potassium has a real and useful action upon epilepsy. By its sedative action upon the whole system, it calms the accessions of madness consecutive to epilepsy, even when the attacks persist. Experiment with an agent which has already produced such fortunate results, should be continued with ardor."

